# A New Pseudolinear Filter for Bearings-Only Tracking without Requirement of Bias Compensation

**DOI:** 10.3390/s21165444

**Published:** 2021-08-12

**Authors:** Shizhe Bu, Aiqiang Meng, Gongjian Zhou

**Affiliations:** 1 Department of Electronic Engineering, Harbin Institute of Technology, Harbin 150001, China; bushizhe@hit.edu.cn (S.B.); maq.0417@foxmail.com (A.M.); 2 Key Laboratory of Marine Environmental Monitoring and Information Processing, Ministry of Industry and Information Technology, Harbin 150001, China

**Keywords:** bearings-only tracking, pseudolinear estimation, correlation analysis, MMSE framework

## Abstract

In bearings-only tracking systems, the pseudolinear Kalman filter (PLKF) has advantages in stability and computational complexity, but suffers from correlation problems. Existing solutions require bias compensation to reduce the correlation between the pseudomeasurement matrix and pseudolinear noise, but incomplete compensation may cause a loss of estimation accuracy. In this paper, a new pseudolinear filter is proposed under the minimum mean square error (MMSE) framework without requirement of bias compensation. The pseudolinear state-space model of bearings-only tracking is first developed. The correlation between the pseudomeasurement matrix and pseudolinear noise is thoroughly analyzed. By splitting the bearing noise term from the pseudomeasurement matrix and performing some algebraic manipulations, their cross-covariance can be calculated and incorporated into the filtering process to account for their effects on estimation. The target state estimation and its associated covariance can then be updated according to the MMSE update equation. The new pseudolinear filter has a stable performance and low computational complexity and handles the correlation problem implicitly under a unified MMSE framework, thus avoiding the severe bias problem of the PLKF. The posterior Cramer–Rao Lower Bound (PCRLB) for target state estimation is presented. Simulations are conducted to demonstrate the effectiveness of the proposed method.

## 1. Introduction

Target tracking has been researched for decades with a wide range of applications in civilian and military areas. It refers to estimate a moving target’s state using the noise-corrupted measurements collected by one or more sensors at fixed locations or on moving platforms [1,2,3,4,5]. The typical measurements include target range, Doppler velocity and bearing angles, while in passive bearings-only tracking (BOT) systems [6,7,8,9,10,11], the sensors listen for signals emitted by a target and only acquire the bearing data.

Bearings-only tracking has been under intensive investigation in recent decades, and the main challenge is the intrinsic nonlinearities in measurement equations. Early research used the extended Kalman filter (EKF) to estimate target state in Cartesian coordinates, but this filter shows poor performance due to premature collapse of the error covariance matrix [12]. Later on, a modified polar coordinate EKF (MPEKF) was developed in [13] to improve the stability. However, both the EKF and MPEKF require good initialization to avoid divergence. The unscented Kalman filter [14] (UKF) and particle filter [15,16,17] (PF) are also applied for bearings-only tracking. The UKF has better estimation performance than the EKF, but still faces the divergence problems. The PF can exhibit a good performance but at the price of heavy computation load.

Another basic and famous recursive Bayesian estimator is the pseudolinear Kalman filter [18] (PLKF). The PLKF solves the bearings-only tracking problem by converting the nonlinear measurement equation to the pseudolinear equation and then applying the Kalman filter (KF) to produce target state estimates. The PLKF is superior in computational complexity and robust to initialization errors compared to the above nonlinear filtering methods [19,20]. However, the pseudomeasurement matrix is a function of the noisy bearing measurements and correlated with the pseudolinear noise. This makes the PLKF exhibit a bias which can be severe in unfavorable geometries and degrades the tracking performance [21].

Several methods have been presented to improve the performance of the PLKF by compensating or reducing the pseudolinear estimation bias. In [22], a modified pseudolinear estimator (MPLE) is developed to reduce the bias by defining the target motion parameters in a new coordinate system related to modified polar coordinates. In [23], a bias-compensated PLKF (BC-PLKF) method is developed to compensate for the bias of the PLKF. The estimate of the cross-term that contains the pseudomeasurement matrix and pseudolinear noise is calculated and then subtracted from the PLKF estimate to generate the final state estimate. The unbiasedness of methods in [22,23] can only be guaranteed under the assumption of small measurement noise, and their performances will be adversely affected at large measurement noise.

The well-known instrumental variable [24] (IV) estimation is also applied for reducing the bias of the PLKF. The essential step of the IV approach is the formulation of the so-called IV matrix, which is statistically independent of the pseudolinear noise and is strongly correlated with pseudomeasurement matrix. Several IV-based estimators are developed in [25,26,27], but they do not have closed-form solutions and require good initialization to guarantee convergence. The methods in [28,29] utilize the bias compensated estimator to construct an IV matrix, and then implement the IV estimation procedure to obtain asymptotically unbiased estimates. However, the correlation between the IV matrix and the pseudomeasurement matrix can be weaken in the presence of large measurement noise and in unfavorable geometries, which can lead to the estimation performance degradation. To maintain a strong correlation between the IV matrix and pseudomeasurement matrix, an IV Kalman filter (IVKF) based on selective-angle-measurement [30] (SAM) strategy is presented in [23], resulting in the SAM-IVKF method. According to the SAM threshold, the IVKF is implemented in the case of small measurement noise, and the BC-PLKF is selected in the large measurement noise. Benefit from the SAM strategy, the SAM-IVKF has a better tracking performance than the BC-PLKF and the IVKF methods, and is robust to the measurement noise and initialisation errors. However, the SAM-IVKF method is a hybrid method, and its theoretical framework is not unified. In addition, the SAM-IVKF method utilizes a empirical scheme to select the SAM threshold, and the threshold values will have a great influence on the tracking performance [31].

The above methods rely on bias compensation to reduce the correlation between the pseudomeasurement matrix and pseudolinear noise, which can improve the performance of the PLKF. As variants of the PLKF, they all show a stable performance and low computational complexity. However, the bias compensation is not always perfect, especially in case of large measurement noise and unfavorable geometries, which will lead to loss of estimation accuracy and consistency.

In this paper, we propose a new pseudolinear filter under the MMSE framework without requirement of bias compensation. Inspired by the methods in [32,33], we make a thorough analysis about the correlations between the pseudomeasurement matrix and pseudolinear noise, and evaluate their impacts on the estimation results. First, the pseudolinear state-space model of bearings-only tracking problems is formulated. Under the MMSE framework, we provide the expression of each step in the filtering process according to its definition. It is found out that in the step of measurement prediction covariance calculation, the correlation between pseudomeasurement matrix and pseudolinear noise will cause the cross-items that contain the two components to be nonzero matrices, which is different from the traditional linear state-space model. Similar situations can be found in the step of calculating the covariance between the state and measurement. Accordingly, we thoroughly analyze the correlation between the pseudomeasurement matrix and pseudolinear noise, and incorporate their cross-covariance into the corresponding processing steps of the filtering process to account for their effects on the estimation results, resulting in the new pseudolinear filter. The proposed method inherits the merits of the PLKF and implicitly handles the correlation under the MMSE framework, which guarantees its low computational complexity and the stable estimation accuracy. The superiority of the proposed method is illustrated by numerical simulations.

The rest of this paper is organized as follows. In Section 2, the bearings-only tracking problem is formulated. In Section 3, the pseudolinear state-space model is constructed and the new pseudolinear filter is presented in detail. The posterior Cramer–Rao lower bound [34,35] (PCRLB) of the state estimation is derived in Section 4. Section 5 presents the simulation results, followed by conclusions in Section 6.

## 2. Problem Formulation

**Figure 1:**
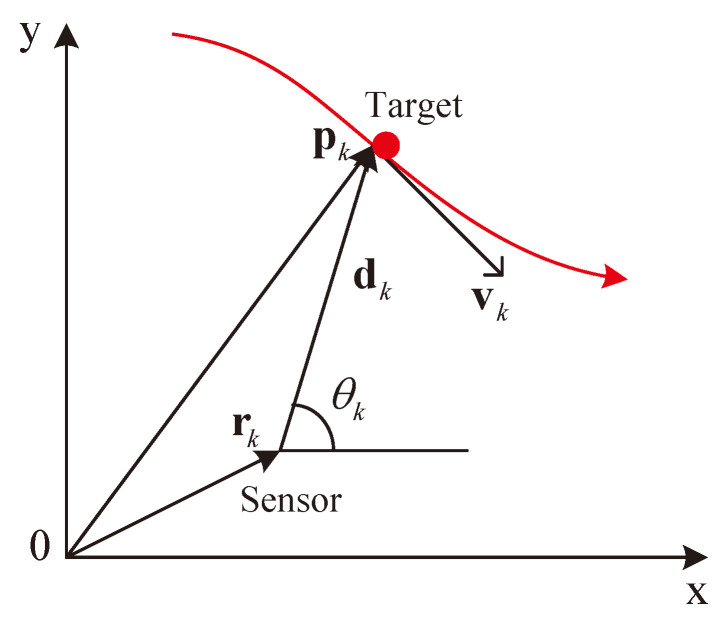
2D bearings-only target tracking geometry.

The problem of bearings-only target tracking by a single moving sensor in the two-dimensional (2D) plane is shown in Figure 1.

As shown in Figure 1, $p_{k}={\left[p_{x,k},\;p_{y,k}\right]}^{T}$ and $v_{k}={\left[v_{x,k},\;v_{y,k}\right]}^{T}$ are the position and velocity of the target at time instant *k*, respectively, which constitute the unknown target state vector $x_{k}={\left[p_{x,k},p_{y,k},v_{x,k},v_{y,k}\right]}^{T}$. $r_{k}={\left[r_{x,k},\;r_{y,k}\right]}^{T}$ is the position of the sensor and assumed to be precisely known at each time instant, and $d_{k}$ is the distance vector pointing from the sensor to the target. We assume that the target follows the nearly constant velocity (NCV) motion [1] in the whole paper. The target state equation is given by

(1) $$x_{k}=Fx_{k-1}+w_{k-1}$$

where F is the target state transition matrix, and $w_{k-1}$ is the zero-mean Gaussian white process noise with known covariance $Q_{k-1}$. The matrices F and $Q_{k-1}$ are given by

(2) $$F=\left[\begin{array}{cccc} 1 & 0 & T & 0\\ 0 & 1 & 0 & T\\ 0 & 0 & 1 & 0\\ 0 & 0 & 0 & 1 \end{array}\right]$$

(3) $$Q_{k-1}=\left[\begin{array}{cccc} \frac{T^{3}}{3}q_{x} & 0 & \frac{T^{2}}{2}q_{x} & 0\\ 0 & \frac{T^{3}}{3}q_{y} & 0 & \frac{T^{2}}{2}q_{y}\\ \frac{T^{2}}{2}q_{x} & 0 & Tq_{x} & 0\\ 0 & \frac{T^{2}}{2}q_{y} & 0 & Tq_{y} \end{array}\right]$$

where *T* is the sampling interval, and $q_{x}$ and $q_{y}$ are power spectral densities of the process noise in the *x*-coordinate and *y*-coordinate, respectively.

According to the geometric relationship in Figure 1, the true bearing angle at time instant *k* is $\theta_{k}=\tan^{-1}\left(p_{y,k}-r_{y,k},\;p_{x,k}-r_{x,k}\right)$, which is corrupted by the independent Gaussian noise $n_{k}$ with zero mean and variance $\sigma_{\theta }^{2}$. The bearing measurement equation is given by

(4) $$\theta_{k}^{z}=\theta_{k}+n_{k}=\tan^{-1}\left(p_{y,k}-r_{y,k},\;p_{x,k}-r_{x,k}\right)+n_{k}$$

where $\theta_{k}^{z}$ is the bearing measurement at time instant *k*. To ensure the target is observable, the sensor needs to maneuver while collecting the bearing measurements [28].

Equations (1) and (4) formulate the state-space model for bearings-only target tracking, and the objective is to estimate the target state using the noise-corrupted bearing measurement at each time instant. Due to the nonlinearity of the bearing measurement equation, the KF cannot be used to obtain the target state estimation. The nonlinear filtering methods such as the EKF and UKF are intuitive solutions for bearings-only target tracking but can lead to instability problems. The PLKF is an attractive alternative due to its stable performance and low computational complexity. However, this method suffers from the correlation problem and results in severe bias problem. Accordingly, it is necessary to find an effective method to solve the PLKF correlation problem, which will be investigated in the next section.

## 3. The New Pseudolinear Filter

In this section, we propose a new pseudolinear filter under the MMSE framework for bearings-only target tracking, which is referred as the pseudolinear-MMSE (PL-MMSE). The PL-MMSE does not require analysis of the PLKF bias caused by the correlation, and performs the bias compensation procedure. Instead, the proposed method evaluates the correlation between the pseudomeasurement matrix and pseudolinear noise, and their cross-covariance is involved in the filtering process to account for their effects on the state and covariance update. The pseudolinear state-space model is presented in the next section, followed by the filtering process of the PL-MMSE.

### 3.1. The Pseudolinear State-Space Model

To be able to apply linear filtering method to the bearings-only tracking, the bearing measurement equation must be linearized. According to the geometry in Figure 1, the bearing measurement equation in (4) is rewritten as

(5) $$\frac{\sin(\theta_{k}^{z}-n_{k})}{\cos(\theta_{k}^{z}-n_{k})}\overset{\Delta }{\;=}\frac{\sin\theta_{k}}{\cos\theta_{k}}\overset{\Delta }{\;=}\frac{p_{y,k}-r_{y,k}}{p_{x,k}-r_{x,k}}$$

where $\sin\theta_{k}=\frac{p_{y,k}-r_{y}}{∥d_{k}∥}$ and $\cos\theta_{k}=\frac{p_{x,k}-r_{x}}{∥d_{k}∥}$. The symbol $∥\cdot ∥$ denotes the Euclidean norm, and the distance vector $d_{k}$ is a function of the target position and sensor position. That is

(6) $$d_{k}=Cx_{k}-r_{k},\;\;\;\;\;\;\;\;C=\left[\begin{array}{c} \begin{array}{cccc} 1 & 0 & 0 & 0 \end{array}\\ \begin{array}{cccc} 0 & 1 & 0 & 0 \end{array} \end{array}\right].$$

We expand (5) according to the triangle formula and have

(7) $$\begin{array}{c} r_{x,k}\sin\theta_{k}^{z}\cos n_{k}-r_{y,k}\cos\theta_{k}^{z}\cos n_{k}=p_{x,k}\sin\theta_{k}^{z}\cos n_{k}-p_{y,k}\cos\theta_{k}^{z}\cos n_{k}\\ \;\;\;\;\;\;\;-\left(p_{x,k}-r_{x,k}\right)\cos\theta_{k}^{z}\sin n_{k}-\left(p_{y,k}-r_{y,k}\right)\sin\theta_{k}^{z}\sin n_{k} \end{array}.$$

Dividing both sides of (7) by $\cos n_{k}$, we have

(8) $$\begin{array}{c} r_{x,k}\sin\theta_{k}^{z}-r_{y,k}\cos\theta_{k}^{z}=p_{x,k}\sin\theta_{k}^{z}-p_{y,k}\cos\theta_{k}^{z}\\ \;\;\;-\left[\left(p_{x,k}-r_{x,k}\right)\cos\theta_{k}^{z}+\left(p_{y,k}-r_{y,k}\right)\sin\theta_{k}^{z}\right]\frac{\sin n_{k}}{\cos n_{k}} \end{array}.$$

Substituting $\theta_{k}^{z}=\theta_{k}+n_{k}$ into the third item in the right side of (8), we have

(9) $$\begin{array}{c} \frac{1}{\cos n_{k}}\left[\left(p_{x,k}-r_{x,k}\right)\cos\theta_{k}^{z}+\left(p_{y,k}-r_{y,k}\right)\sin\theta_{k}^{z}\right]\\ =\left(p_{x,k}-r_{x,k}\right)\left(\cos\theta_{k}-\sin\theta_{k}\tan n_{k}\right)+\left(p_{y,k}-r_{y,k}\right)\left(\sin\theta_{k}+\cos\theta_{k}\tan n_{k}\right)\\ \begin{array}{c} =\left(p_{x,k}-r_{x,k}\right)\left(\frac{p_{x,k}-r_{x,k}}{∥d_{k}∥}-\frac{p_{y,k}-r_{y,k}}{∥d_{k}∥}\tan n_{k}\right)\\ +\left(p_{y,k}-r_{y,k}\right)\left(\frac{p_{y,k}-r_{y,k}}{∥d_{k}∥}+\frac{p_{x,k}-r_{x,k}}{∥d_{k}∥}\tan n_{k}\right) \end{array}\\ =\frac{{\left(p_{x,k}-r_{x,k}\right)}^{2}+{\left(p_{y,k}-r_{y,k}\right)}^{2}}{∥d_{k}∥}=∥d_{k}∥ \end{array}.$$

After the manipulations in (5)–(9), the nonlinear bearing measurement equation in (4) is converted into a pseudolinear function of the state vector $x_{k}$

(10) $$z_{k}=H_{k}x_{k}+\eta_{k}$$

where the measurement $z_{k}$, the pseudomeasurement matrix $H_{k}$, and the pseudolinear noise $\eta_{k}$ are, respectively, given by

(11) $$z_{k}=r_{x,k}\sin\theta_{k}^{z}-r_{y,k}\cos\theta_{k}^{z}.$$

(12) $$H_{k}=\left[\sin\theta_{k}^{z},\;-\cos\theta_{k}^{z},\;0,\;0\right].$$

(13) $$\eta_{k}=-∥d_{k}∥\sin n_{k}.$$

The bearing noise $n_{k}$ is assumed to be zero-mean Gaussian white variable. In this case, one has [36]

(14a) $$E\left[\cos n_{k}\right]=e^{-\frac{\sigma_{\theta }^{2}}{2}}.$$

(14b) $$E[\sin n_{k}]=0.$$

(14c) $$E\left[\cos^{2}n_{k}\right]=\frac{1+e^{-2\sigma_{\theta }^{2}}}{2}.$$

(14d) $$E\left[\sin^{2}n_{k}\right]=\frac{1-e^{-2\sigma_{\theta }^{2}}}{2}.$$

(14e) $$E[\cos n_{k}\sin n_{k}]=0.$$

Accordingly, the mean $\mu_{k}$ and the variance $R_{k}$ of the pseudolinear noise $\eta_{k}$ are

(15a) $$\mu_{k}=E\left[\eta_{k}\right]=-∥d_{k}∥E\left[\sin n_{k}\right]=0.$$

(15b) $$R_{k}=E\left[\eta_{k}^{2}\right]={∥d_{k}∥}^{2}E\left[\sin^{2}n_{k}\right]=\frac{1-e^{-2\sigma_{\theta }^{2}}}{2}{∥d_{k}∥}^{2}.$$

Using the pseudolinear measurement, Equation (10), the pseudolinear state-space model for bearings-only target tracking can be described as

(16a) $$x_{k}=Fx_{k-1}+w_{k-1}.$$

(16b) $$z_{k}=H_{k}x_{k}+\eta_{k}.$$

The pseudomeasurement matrix $H_{k}$ and the pseudolinear noise $\eta_{k}$ in (16b) both are functions of bearing noise $n_{k}$, which causes $H_{k}$ and $\eta_{k}$ to be correlated. This leads to the significant difference between the pseudolinear state-space model (16a)−(16b) and the traditional linear state-space model. The PLKF ignores the correlation between $H_{k}$ and $\eta_{k}$, and directly applies the KF to estimate the target state according to the pseudolinear state-space model. To solve this problem, we analyze the correlation between $H_{k}$ and $\eta_{k}$, and incorporate their cross-covariance into the filtering process under the MMSE framework to account for the effects on the estimation results.

### 3.2. Filtering Process

In this subsection, the filtering process of the PL-MMSE is presented under the MMSE framework, including the prediction stage, covariance calculation stage and update stage.

#### 3.2.1. Prediction Stage

According to Equation (16a), the one-step predicted state is

(17) $${\hat{x}}_{k\left|k-1\right.}=E\left[x_{k}\left|Z^{k-1}\right.\right]=E\left[Fx_{k-1}+w_{k-1}\left|Z^{k-1}\right.\right]=F{\hat{x}}_{k-1\left|k-1\right.}$$

where $Z^{k-1}$ denotes the sequence of measurements available at time instant k−1. Subtracting the above from (16a) yields the state prediction error

(18) $${\tilde{x}}_{k\left|k-1\right.}=x_{k}-{\hat{x}}_{k\left|k-1\right.}=F{\tilde{x}}_{k-1\left|k-1\right.}+w_{k-1}.$$

The state prediction covariance is

(19) $$\begin{array}{c} P_{k\left|k-1\right.}=E\left[{\tilde{x}}_{k\left|k-1\right.}\cdot {\tilde{x}}_{k\left|k-1\right.}^{T}\left|Z^{k-1}\right.\right]=E\left[\left(F{\tilde{x}}_{k-1\left|k-1\right.}+w_{k-1}\right){\left(F{\tilde{x}}_{k-1\left|k-1\right.}+w_{k-1}\right)}^{T}\left|Z^{k-1}\right.\right]\\ \;\;\;\;\;\;\;\;=FP_{k-1\left|k-1\right.}F^{T}+Q_{k-1} \end{array}$$

where the cross-items contains ${\tilde{x}}_{k-1\left|k-1\right.}$ and $w_{k-1}$ vanish since the process noise $w_{k-1}$ is independent zero-mean Gaussian white variable.

The predicted measurement ${\hat{z}}_{k\left|k-1\right.}$ is obtained by taking the expected value of (16b) conditioned on $Z^{k-1}$. That is,

(20) $$\begin{array}{c} {\hat{z}}_{k\left|k-1\right.}=E\left[z_{k}\left|Z^{k-1}\right.\right]=E\left[H_{k}x_{k}+\eta_{k}\left|Z^{k-1}\right.\right]\\ \;\;\;\;\;\;\;\;=H_{k}\cdot E\left[x_{k}\left|Z^{k-1}\right.\right]+E\left[\eta_{k}\left|Z^{k-1}\right.\right]=H_{k}{\hat{x}}_{k\left|k-1\right.} \end{array}$$

where $H_{k}$ is a deterministic vector according to (12), which can be pulled out from the expectation operator. Subtracting the above from (16b) yields the measurement prediction error

(21) $${\tilde{z}}_{k\left|k-1\right.}=z_{k}-{\hat{z}}_{k\left|k-1\right.}=H_{k}x_{k}+\eta_{k}-H_{k}{\hat{x}}_{k\left|k-1\right.}=H_{k}{\tilde{x}}_{k\left|k-1\right.}+\eta_{k}.$$

#### 3.2.2. Covariance Calculation Stage

In this part, we will present the steps to calculate the measurement prediction covariance $P_{zz}$, and the covariance $P_{xz}$ between the state and measurement. As discussed in Section 1, the correlation between $H_{k}$ and $\eta_{k}$ will cause the cross-terms in $P_{zz}$ that contains the two components to be nonzero matrices. Similar situations can be found in $P_{xz}$. Accordingly, we will first present the expression of $P_{xz}$ and $P_{zz}$ according to their definitions. By splitting the bearing noise term from the pseudomeasurement matrix and performing some algebraic manipulations, the estimates of the cross-terms that contains $H_{k}$ and $\eta_{k}$ can be calculated.

The measurement prediction covariance is

(22) $$\begin{array}{cc} P_{zz}=E\left[{\tilde{z}}_{k\left|k-1\right.}{\tilde{z}}_{k\left|k-1\right.}^{T}\right] & =E\left[\left(H_{k}{\tilde{x}}_{k\left|k-1\right.}+\eta_{k}\right){\left(H_{k}{\tilde{x}}_{k\left|k-1\right.}+\eta_{k}\right)}^{T}\right]\\ \; & =E[H_{k}{\tilde{x}}_{k\left|k-1\right.}{\tilde{x}}_{k\left|k-1\right.}^{T}H_{k}^{T}+H_{k}{\tilde{x}}_{k\left|k-1\right.}\eta_{k}^{T}+\eta_{k}{\tilde{x}}_{k\left|k-1\right.}^{T}H_{k}^{T}+\eta_{k}^{2}]\\ \; & =H_{k}P_{k|k-1}H_{k}^{T}+E\left[H_{k}{\tilde{x}}_{k\left|k-1\right.}\eta_{k}^{T}\right]+E\left[\eta_{k}{\tilde{x}}_{k\left|k-1\right.}^{T}H_{k}^{T}\right]+R_{k} \end{array}$$

where $H_{k}$ and $\eta_{k}$ both are functions of bearing noise $n_{k}$ and correlated with each other, causing the second and third items in (22) to be nonzero matrices, which should be calculated as follows.

First, split $n_{k}$ out of $H_{k}$. That is,

(23) $$\begin{array}{c} H_{k}=\left[\sin\left(\theta_{k}+n_{k}\right),\;-\cos\left(\theta_{k}+n_{k}\right),\;0,\;0\right]\\ \;\;\;\;\;\;=\left[\left(\sin\theta_{k}\cos n_{k}+\cos\theta_{k}\sin n_{k}\right),\;\left(-\cos\theta_{k}\cos n_{k}+\sin\theta_{k}\sin n_{k}\right),\;0,\;0\right]\\ \;\;\;\;\;\;=\cos n_{k}\left[\sin\theta_{k},\;-\cos\theta_{k},\;0,\;0\right]+\sin n_{k}\left[\cos\theta_{k},\;\sin\theta_{k},\;0,\;0\right] \end{array}.$$

For simplicity, we denote

(24a) $$H_{1,k}=\left[\begin{array}{cccc} \sin\theta_{k} & -\cos\theta_{k} & 0 & 0 \end{array}\right].$$

(24b) $$H_{2,k}=\left[\begin{array}{cccc} \cos\theta_{k} & \sin\theta_{k} & 0 & 0 \end{array}\right].$$

and (23) can be rewritten as

(25) $$H_{k}=\cos n_{k}H_{1,k}+\sin n_{k}H_{2,k}.$$

Second, we substitute $\sin\theta_{k}=\frac{p_{y,k}-r_{y}}{∥d_{k}∥}$ and $\cos\theta_{k}=\frac{p_{x,k}-r_{x}}{∥d_{k}∥}$ into (25). Since the target position $p_{k}={\left[p_{x,k},\;p_{y,k}\right]}^{T}$ is unavailable in practice, the approximate forms $p_{x,k}={\hat{x}}_{k\left|k-1\right.}\left(1\right)+{\tilde{x}}_{k\left|k-1\right.}\left(1\right)$ and $p_{y,k}={\hat{x}}_{k\left|k-1\right.}\left(2\right)+{\tilde{x}}_{k\left|k-1\right.}\left(2\right)$ are used as substitutes.

Therefore, the second cross-term of (22), i.e., $E[H_{k}{\tilde{x}}_{k\left|k-1\right.}\eta_{k}^{T}]$, can be rewritten as

(26) $$\begin{array}{c} E\left[H_{k}{\tilde{x}}_{k\left|k-1\right.}\eta_{k}^{T}\right]=E\left[\left(\cos n_{k}H_{1,k}+\sin n_{k}H_{2,k}\right){\tilde{x}}_{k\left|k-1\right.}\left(-∥d_{k}∥\sin n_{k}\right)\right]\\ =E\left[-\sin^{2}n_{k}∥d_{k}∥H_{2,k}{\tilde{x}}_{k\left|k-1\right.}\right]\\ =E\left[-\sin^{2}n_{k}\right]E\left\{∥d_{k}∥\left[\cos\theta_{k},\;\sin\theta_{k},\;0,\;0\right]{\tilde{x}}_{k\left|k-1\right.}\right\}\\ \begin{array}{c} =\frac{e^{-2\sigma_{\theta }^{2}}-1}{2}\\ \times E\left\{∥d_{k}∥\left[\frac{{\hat{x}}_{k\left|k-1\right.}\left(1\right)+{\tilde{x}}_{k\left|k-1\right.}\left(1\right)-r_{x,k}}{∥d_{k}∥},\;\frac{{\hat{x}}_{k\left|k-1\right.}\left(2\right)+{\tilde{x}}_{k\left|k-1\right.}\left(2\right)-r_{y,k}}{∥d_{k}∥},\;0,\;0\right]{\tilde{x}}_{k\left|k-1\right.}\right\} \end{array}\\ =\frac{e^{-2\sigma_{\theta }^{2}}-1}{2}E\left\{\left[{\hat{x}}_{k\left|k-1\right.}\left(1\right)+{\tilde{x}}_{k\left|k-1\right.}\left(1\right)-r_{x,k},\;{\hat{x}}_{k\left|k-1\right.}\left(2\right)+{\tilde{x}}_{k\left|k-1\right.}\left(2\right)-r_{y,k},\;0,\;0\right]{\tilde{x}}_{k\left|k-1\right.}\right\}\\ =\frac{e^{-2\sigma_{\theta }^{2}}-1}{2}\left[P_{k\left|k-1\right.}\left(1,1\right)+P_{k\left|k-1\right.}\left(2,2\right)\right] \end{array}$$

where $P_{k\left|k-1\right.}\left(j,j\right)$ represents the element located at the jth row and jth column of $P_{k\left|k-1\right.}$.

The third item $E[\eta_{k}{\tilde{x}}_{k\left|k-1\right.}^{T}H_{k}^{T}]$ of (22) is the transpose of $E[H_{k}{\tilde{x}}_{k\left|k-1\right.}\eta_{k}^{T}]$ and given by

(27) $$E\left[\eta_{k}{\tilde{x}}_{k\left|k-1\right.}^{T}H_{k}^{T}\right]=\frac{e^{-2\sigma_{\theta }^{2}}-1}{2}\left[P_{k\left|k-1\right.}\left(1,1\right)+P_{k\left|k-1\right.}\left(2,2\right)\right].$$

The measurement noise variance $R_{k}$ in (22) is $\frac{1-e^{-2\sigma_{\theta }^{2}}}{2}{∥d_{k}∥}^{2}$ according to (15b), where the distance vector $d_{k}=Cx_{k}-r_{k}$ is unavailable due to the unknown state $x_{k}$. To overcome this, the predicted distance vector ${\hat{d}}_{k\left|k-1\right.}=C{\hat{x}}_{k\left|k-1\right.}-r_{k}$, approximated from $d_{k}$, is used as a substitute.

Substituting all the required matrices into (22), the measurement prediction covariance can be rewritten as

(28) $$P_{zz}=H_{k}P_{k|k-1}H_{k}^{T}+\left(e^{-2\sigma_{\theta }^{2}}-1\right)\left[P_{k|k-1}\left(1,1\right)+P_{k|k-1}\left(2,2\right)\right]+\frac{1-e^{-2\sigma_{\theta }^{2}}}{2}{∥{\hat{d}}_{k\left|k-1\right.}∥}^{2}.$$

Similar to the derivation of $P_{zz}$, the covariance $P_{xz}$ between the state and measurement is given by

(29) $$\begin{array}{c} P_{xz}=E\left[{\tilde{x}}_{k\left|k-1\right.}{\tilde{z}}_{k\left|k-1\right.}^{T}\right]=E\left[{\tilde{x}}_{k\left|k-1\right.}{\left(H_{k}{\tilde{x}}_{k\left|k-1\right.}+\eta_{k}\right)}^{T}\right]\\ \;\;\;\;\;=E\left[{\tilde{x}}_{k\left|k-1\right.}{\tilde{x}}_{k\left|k-1\right.}^{T}{\left(\cos n_{k}H_{1,k}+\sin n_{k}H_{2,k}\right)}^{T}-\sin n_{k}∥d_{k}∥{\tilde{x}}_{k\left|k-1\right.}\right]\\ \;\;\;\;\;=E\left[\cos n_{k}\right]E\left[{\tilde{x}}_{k\left|k-1\right.}{\tilde{x}}_{k\left|k-1\right.}^{T}H_{1,k}^{T}\right]=e^{-\frac{\sigma_{\theta }^{2}}{2}}P_{k\left|k-1\right.}H_{1,k}^{T} \end{array}$$

where $H_{1,k}$ relies on the true bearing angle $\theta_{k}$ as shown in (24a), which is unavailable in practice. To make the result useful, we replace the unknown true bearing angle $\theta_{k}$ with the predicted angle ${\hat{\theta }}_{k\left|k-1\right.}$ computed from ${\hat{x}}_{k|k-1}$. That is,

(30) $${\hat{\theta }}_{k\left|k-1\right.}=\tan^{-1}\left({\hat{x}}_{k\left|k-1\right.}\left(2\right)-r_{y,k},\;{\hat{x}}_{k\left|k-1\right.}\left(1\right)-r_{x,k}\right).$$

(31) $${\hat{H}}_{1,k}=\left[\sin{\hat{\theta }}_{k\left|k-1\right.},\;-\cos{\hat{\theta }}_{k\left|k-1\right.},\;0,\;0\right].$$

We can rewrite the covariance between the state and measurement as

(32) $$P_{xz}=e^{-\frac{\sigma_{\theta }^{2}}{2}}P_{k\left|k-1\right.}{\hat{H}}_{1,k}^{T}.$$

#### 3.2.3. Update Stage

Based on the above results, the state and covariance update equations at time instant *k* under the MMSE framework can be given by

(33) $${\hat{x}}_{k\left|k\right.}={\hat{x}}_{k\left|k-1\right.}+P_{xz}P_{zz}^{-1}\left(z_{k}-{\hat{z}}_{k\left|k-1\right.}\right).$$

(34) $$P_{k\left|k\right.}=P_{k|k-1}-P_{xz}P_{zz}^{-1}P_{xz}^{T}.$$

The filtering process of the PL-MMSE is carried out in the pseudolinear state-space model, thereby ensuring the low complexity and stable performance like the PLKF method. Meanwhile, the PL-MMSE incorporates the cross-covariance between the pseudomeasurement matrix and pseudolinear noise into its filtering process, so the correlation problem can be handled implicitly under a unified MMSE estimation framework. This can avoid the severe bias in estimation results of the PLKF. Additionally, the PL-MMSE does not require bias compensation like the previous reduced-bias methods, thus avoiding the loss of estimation accuracy caused by the possible incomplete compensation.

## 4. Lower Bound of Performance

Since the bearing measurement, Equation (4), is nonlinear, the optimal solution to the bearings-only tracking problem cannot be derived analytically. A theoretical lower bound of performance would be helpful to assess the level of approximation introduced by the proposed method. The PCRLB on the variance of estimation error provides the performance limit for any unbiased estimator of a fixed parameter, which is derived briefly as follows.

The lower bound on the estimation error is determined by the Fisher information matrix $J_{k}$, and the covariance of ${\hat{x}}_{k\left|k\right.}$ is bounded by

(35) $$E\left\{\left({\hat{x}}_{k\left|k\right.}-x_{k}\right){\left({\hat{x}}_{k\left|k\right.}-x_{k}\right)}^{T}\right\}\geq J_{k}^{-1}=\mathrm{PCRL}B_{x_{k}}.$$

The general frame work for derivation of PCRLB of an unbiased estimator for nonlinear discrete-time system is described in [34], and the information matrix can be calculated by recursion [34,37]

(36) $$J_{k}=D_{k-1}^{22}-D_{k-1}^{21}{\left[J_{k-1}+D_{k-1}^{11}\right]}^{-1}D_{k-1}^{12}$$

where

(37a) $$D_{k-1}^{11}=F^{T}Q_{k-1}^{-1}F$$

(37b) $$D_{k-1}^{12}=-F^{T}Q_{k-1}^{-1}={\left[D_{k-1}^{21}\right]}^{T}$$

(37c) $$D_{k-1}^{22}=Q_{k-1}^{-1}+E_{x_{k}}\left\{\left[\nabla_{x_{k}}{\theta_{k}}^{T}\right]{\left[\sigma_{\theta }^{2}\right]}^{-1}{\left[\nabla_{x_{k}}{\theta_{k}}^{T}\right]}^{T}\right\}$$

where the expectation $E\left\{\cdot \right\}$ in (37c) is taken over $x_{k}$, $\nabla_{x_{k}}$ is the gradient operator, and ${\left[\nabla_{x_{k}}{\theta_{k}}^{T}\right]}^{T}$ is the Jacobian matrix of $\theta_{k}$ evaluated at the true target state $x_{k}$. For simplicity, we denote $V_{k}={\left[\nabla_{x_{k}}{\theta_{k}}^{T}\right]}^{T}$, which is given by

(38) $$V_{k}=\left[\frac{\partial \theta_{k}}{\partial p_{x,k}},\;\frac{\partial \theta_{k}}{\partial p_{y,k}},\;\frac{\partial \theta_{k}}{\partial v_{x,k}},\;\frac{\partial \theta_{k}}{\partial v_{x,k}}\right]$$

and the entries of $V_{k}$ are

(39a) $$\frac{\partial \theta_{k}}{\partial p_{x,k}}=-\frac{p_{y,k}-r_{y,k}}{{∥p_{k}-r_{k}∥}^{2}}.$$

(39b) $$\frac{\partial \theta_{k}}{\partial p_{y,k}}=\frac{p_{x,k}-r_{x,k}}{{∥p_{k}-r_{k}∥}^{2}}.$$

(39c) $$\frac{\partial \theta_{k}}{\partial v_{x,k}}=\frac{\partial \theta_{k}}{\partial v_{y,k}}=0.$$

Using the matrix inversion lemma, we can show that (36) and (37a)−(37c) are equivalent to the following recursion:

(40) $$J_{k}={\left[Q_{k-1}+{\mathrm{FJ}}_{k-1}^{-1}F^{T}\right]}^{-1}+\frac{1}{\sigma_{\theta }^{2}}E_{x_{k}}\left\{V_{k}^{T}V_{k}\right\}.$$

The PCRLBs of the target state components are calculated as the corresponding diagonal elements of the inverse information matrix

(41) $$\mathrm{PCRL}B_{x_{k}}\left(j,j\right)={\left[{J_{k}}^{-1}\right]}_{jj}$$

where $\mathrm{PCRL}B_{x_{k}}\left(j,j\right)$ denotes the PCRLB of the jth component of the state $x_{k}$, and ${\left[\cdot \right]}_{jj}$ represents the element located at the jth row and jth column of a matrix.

The recursion in (40) can be implemented based on Monte Carlo simulation averaging over multiple realizations of the target trajectory. Given the initial information matrix, we can calculate the PCRLB through the recursion in (40). In practice, the recursion can be initialized with the inverse of the initial covariance matrix of the filtering method as $J_{k}={P_{1|1}}^{-1}$, which will be presented in Section 5.2.

## 5. Simulation Results

Simulations and performance comparisons are presented in this section to evaluate the effectiveness of the proposed method. The proposed PL-MMSE method is compared with the existing PLKF [18], BC-PLKF [23] and SAM-IVKF [23] methods. In the simulations, *M* = 10,000 Monte Carlo runs are carried out on the given experiment, and the number of the sampling time instants is set as N=150 in each run.

### 5.1. Performance Metrics

Several performance metrics are introduced in this subsection to evaluate the performance of these methods, including the root mean square errors (RMSEs), the bias norms (BNorms) and the normalized estimation error squared [1] (NEES). The PCRLB is also used as the performance benchmark to quantify the best achievable accuracy.

The position and velocity RMSEs are, respectively, defined by

(42a) $${RMSE}_{k}^{p}=\sqrt{\frac{1}{M}\sum_{i=1}^{M}{∥{\hat{p}}_{k\left|k\right.}^{i}-p_{k}^{i}∥}^{2}}$$

(42b) $${RMSE}_{k}^{v}=\sqrt{\frac{1}{M}\sum_{i=1}^{M}{∥{\hat{v}}_{k\left|k\right.}^{i}-v_{k}^{i}∥}^{2}}$$

where $p_{k}^{i}={\mathrm{Cx}}_{k}^{i}$ and ${\hat{p}}_{k\left|k\right.}^{i}={C\hat{x}}_{k\left|k\right.}^{i}$ are the true and estimated target positions at the *i*th Monte Carlo run at time instant *k*, $v_{k}^{i}={\mathrm{Dx}}_{k}^{i}$ and ${\hat{v}}_{k\left|k\right.}^{i}={D\hat{x}}_{k\left|k\right.}^{i}$ are the true and estimated target velocities, where

(43) $$D=\left[\begin{array}{c} \begin{array}{cccc} 0 & 0 & 1 & 0 \end{array}\\ \begin{array}{cccc} 0 & 0 & 0 & 1 \end{array} \end{array}\right].$$

The position and velocity BNorms are, respectively, defined by

(44a) $${BNorm}_{k}^{p}=∥\frac{1}{M}\sum_{i=1}^{M}\left({\hat{p}}_{k\left|k\right.}^{i}-p_{k}^{i}\right)∥.$$

(44b) $${BNorm}_{k}^{v}=∥\frac{1}{M}\sum_{i=1}^{M}\left({\hat{v}}_{k\left|k\right.}^{i}-v_{k}^{i}\right)∥.$$

The position and velocity PCRLBs at time instant *k* are the square root of the sum of the corresponding elements on the diagonal of $\mathrm{PCRL}B_{x_{k}}$, which are, respectively, given by

(45a) $${PCRLB}_{k}^{p}=\sqrt{\mathrm{PCRL}B_{x_{k}}\left(1,1\right)+\mathrm{PCRL}B_{x_{k}}\left(2,2\right)}$$

(45b) $${PCRLB}_{k}^{v}=\sqrt{\mathrm{PCRL}B_{x_{k}}\left(3,3\right)+\mathrm{PCRL}B_{x_{k}}\left(4,4\right)}$$

where $\mathrm{PCRL}B_{x_{k}}\left(j,j\right)$ has been defined in (41).

To compare the average performance of these methods at different noise levels, the time-averaged RMSEs, BNorms and PCRLBs are also utilized. The time-averaged RMSEs, BNorms and PCRLBs are, respectively, defined by

(46a) $${RMSE}_{avg}^{p}=\sqrt{\frac{1}{MU}\sum_{i=1}^{M}\sum_{k=L}^{N}{∥{\hat{p}}_{k\left|k\right.}^{i}-p_{k}^{i}∥}^{2}}$$

(46b) $${\mathrm{BNorm}}_{avg}^{p}=\frac{1}{U}\sum_{k=L}^{N}\left(∥\frac{1}{M}\sum_{i=1}^{M}\left({\hat{p}}_{k\left|k\right.}^{i}-p_{k}^{i}\right)∥\right)$$

(46c) $${RMSE}_{avg}^{v}=\sqrt{\frac{1}{MU}\sum_{i=1}^{M}\sum_{k=L}^{N}{∥{\hat{v}}_{k\left|k\right.}^{i}-v_{k}^{i}∥}^{2}}$$

(46d) $${\mathrm{BNorm}}_{avg}^{v}=\frac{1}{U}\sum_{k=L}^{N}\left(∥\frac{1}{M}\sum_{i=1}^{M}\left({\hat{v}}_{k\left|k\right.}^{i}-v_{k}^{i}\right)∥\right)$$

(46e) $${PCRLB}_{avg}^{p}=\sqrt{\mathrm{PCRL}B_{avg}\left(1,1\right)+\mathrm{PCRL}B_{avg}\left(2,2\right)}$$

(46f) $${PCRLB}_{avg}^{v}=\sqrt{\mathrm{PCRL}B_{avg}\left(3,3\right)+\mathrm{PCRL}B_{avg}\left(4,4\right)}$$

where

(47) $$\mathrm{PCRL}B_{avg}=\frac{1}{U}\sum_{k=L}^{N}\mathrm{PCRL}B_{x_{k}}.$$

Here, U=N−L+1, where *L* is an offset parameter to make the time-averaged performance metrics unaffected by the initial estimation errors. We set *L* = 60 in the simulations.

### 5.2. Simulation Parameters

**Figure 2:**
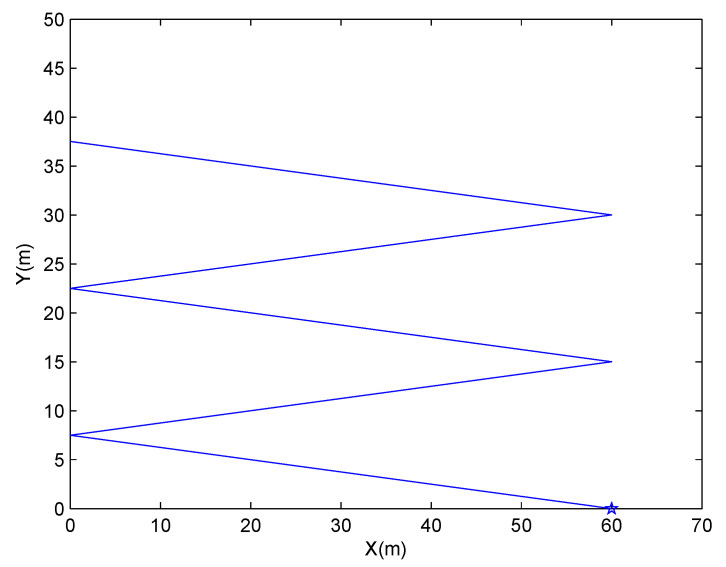
Sensor trajectory.

We consider a single target tracking problem with a single bearings-only sensor in the 2D plane. To perform an objective and fair performance comparison with the SAM-IVKF, we use the same target-observer geometry as in [23]. Specifically, the trajectory of the sensor is five constant-velocity legs with the end position of each leg set to ${\left[60,0\right]}^{T}\;m$, ${\left[0,7.5\right]}^{T}\;m$, ${\left[60,15\right]}^{T}\;m$, ${\left[0,22.5\right]}^{T}\;m$, ${\left[60,30\right]}^{T}\;m$ and ${\left[0,77.5\right]}^{T}\;m$. The sensor trajectory is depicted in Figure 2 and its initial position is marked with Pentagram. The sensor collects bearing measurements at regular time instants $t_{k}=kT,\;k\in \left\{1,2,\ldots ,150\right\}$ with the sampling interval being T=0.1s. The measurement noise $n_{k}$ is assumed to be i.i.d. with known variance $\sigma_{\theta }^{2}$, whose value will be given next. The moving target takes a constant velocity ${\left[0,12\right]}^{T}\;m/s$ starting from the position ${\left[30,42\right]}^{T}\;m$. The power spectral densities of the process noise are set to $q_{x}=q_{y}=0.2\;m^{2}/s^{3}$. In the simulations, track initialization is obtained as in [38] by generating a target state estimate ${\hat{x}}_{1\left|1\right.}$ from a Gaussian distribution around the true target state $x_{1}$ with the covariance given by $P_{1\left|1\right.}=\rho^{2}\mathrm{diag}\left(2.6^{2},2.6^{2},0.26^{2},0.26^{2}\right)$. The variable ρ controls the level of track initialization error and is used to evaluate the performance of the tracking algorithms with respect to initialization error levels.

### 5.3. Tracking Performance

The tracking performance of the proposed PL-MMSE, the PLKF, the BC-PLKF and the SAM-PLKF for different measurement noise levels is compared in this subsection. In practice, the initialization error ρ is often proportional to the measurement noise $\sigma_{\theta }$, and their values in each level are given in Table 1. According to [23], the performance of the SAM-IVKF depends on the selection of the threshold κ. Therefore, the SAM-IVKF under the thresholds κ=4, 3 and 2 is compared with the proposed PL-MMSE method.

**Figure 3:**
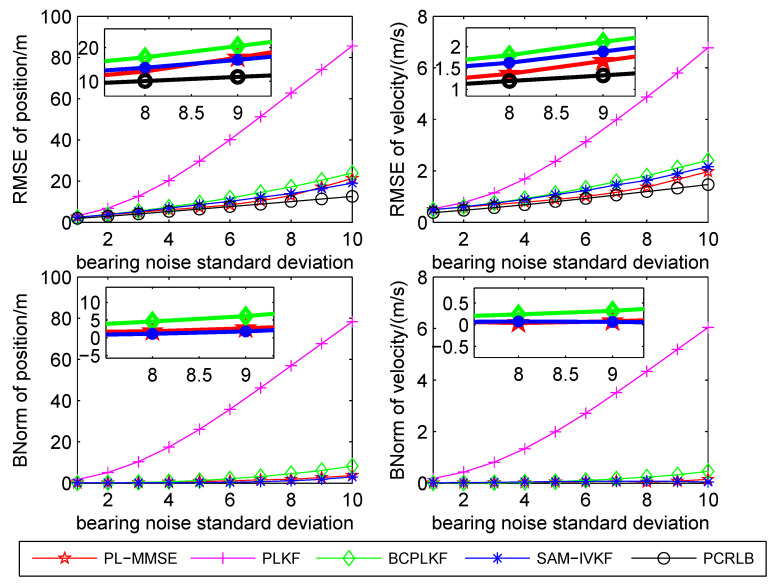
Time-averaged RMSEs and BNorms versus bearing noises for the PLKF, the BC-PLKF and the SAM-IVKF with κ=4, as well as, the proposed PL-MMSE.

**Figure 4:**
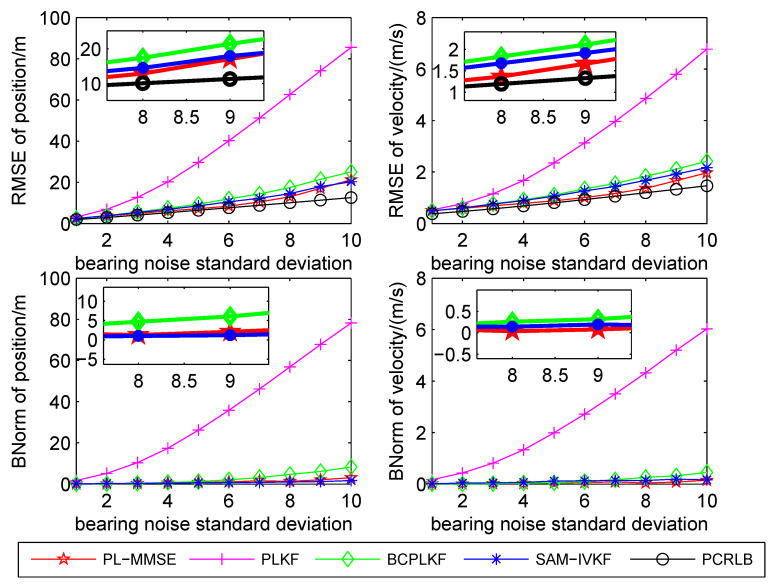
Time-averaged RMSEs and BNorms versus bearing noises for the PLKF, the BC-PLKF and the SAM-IVKF with κ=3, as well as, the proposed PL-MMSE.

**Figure 5:**
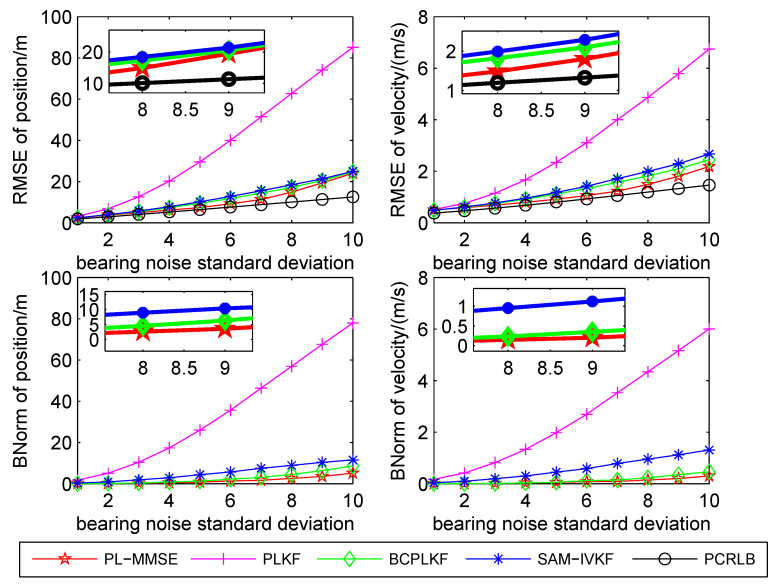
Time-averaged RMSEs and BNorms versus bearing noises for the PLKF, the BC-PLKF and the SAM-IVKF with κ=2, as well as, the proposed PL-MMSE.

**Figure 6:**
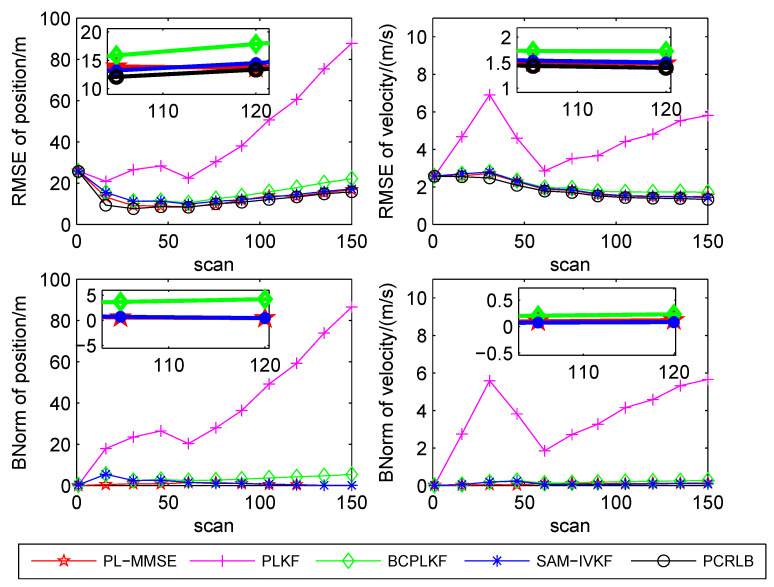
RMSEs and BNorms versus time *k* for $\sigma_{\theta }\;=\;7^{\circ }$ for the PLKF, the BC-PLKF and the SAM-IVKF with κ=4, as well as, the proposed PL-MMSE.

**Figure 7:**
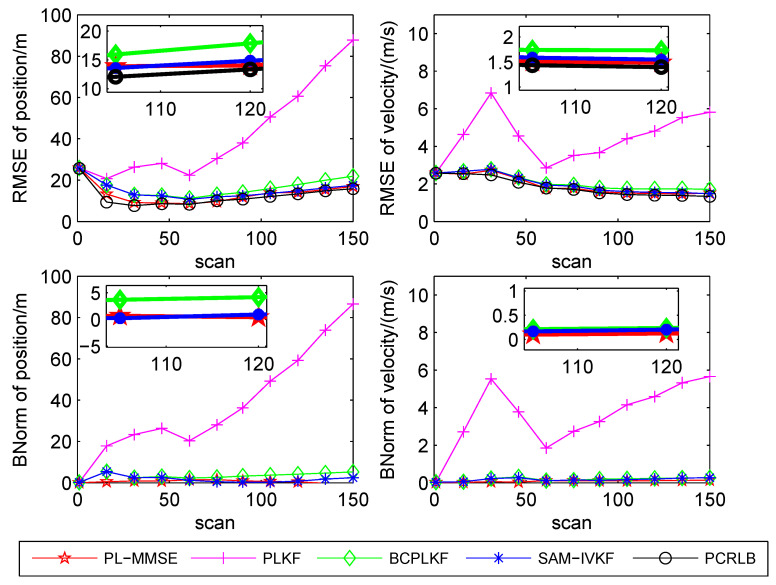
RMSEs and BNorms versus time *k* for $\sigma_{\theta }\;=\;7^{\circ }$ for the PLKF, the BC-PLKF and the SAM-IVKF with κ=3, as well as, the proposed PL-MMSE.

**Figure 8:**
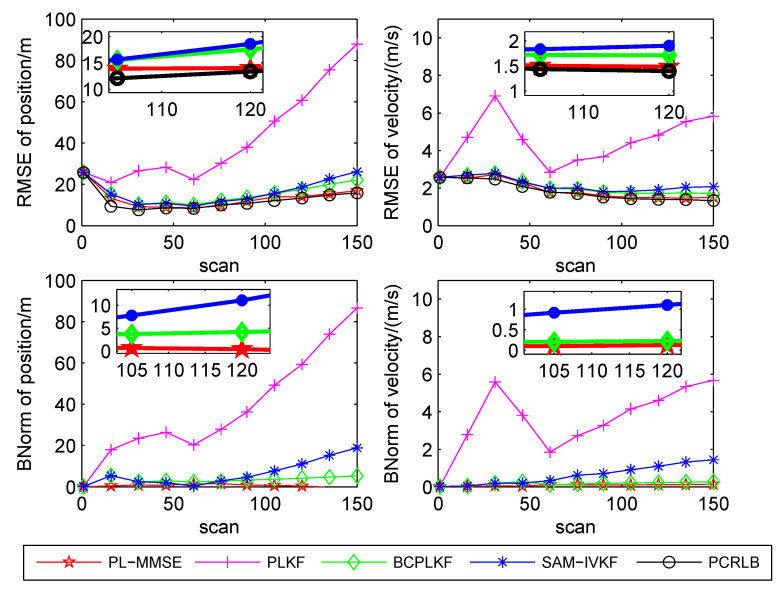
RMSEs and BNorms versus time *k* for $\sigma_{\theta }\;=\;7^{\circ }$ for the PLKF, the BC-PLKF and the SAM-IVKF with κ=2, as well as, the proposed PL-MMSE.

The time-averaged RMSEs and BNorms of the target position and velocity estimates versus the bearing noise standard deviations are shown in Figure 3, Figure 4 and Figure 5. The SAM-IVKF under the thresholds κ=4, 3 and 2 are plotted in this figures, respectively. Additionally, the evolution of RMSEs and BNorms of the target position and velocity estimates for $\sigma_{\theta }=7^{\circ }$ and κ=4,3 and 2 are presented in Figure 6, Figure 7 and Figure 8, respectively. The PCRLB is used to quantify the best achievable accuracy.

As shown in Figure 3, Figure 4 and Figure 5, the PLKF suffers from severe bias problem and provides unsatisfactory RMSEs and BNorms performances at both small and large bearing noise levels. As the noise standard deviation $\sigma_{\theta }$ increases, the time-averaged RMSEs and BNorms performances of the PLKF deteriorate rapidly. The previously developed BC-PLKF and SAM-IVKF methods compensate for the bias and have a performance improvement compared to the PLKF. The BC-PLKF shows a good performance at small noise levels, but the bias cannot be completely compensated under large bearing noise ($\sigma_{\theta }\geq 7^{\circ }$), which leads to a decrease in performance. Benefit from the SAM strategy, the time-averaged RMSEs and BNorms of the SAM-IVKF are lower than those of the BC-PLKF under the best choice of the threshold, i.e., κ=4, as shown in Figure 3. However, the performance of the SAM-IVKF may vary greatly as the SAM thresholds change, and its performance is even worse than the BC-PLKF under the threshold κ=2, as shown in Figure 5.

The proposed PL-MMSE method also has a significant performance improvement compared to the PLKF, and shows good stability at both small and large bearing noise levels. The target state RMSEs of the PL-MMSE approach the PCRLB. Compared with the BC-PLKF, the PL-MMSE has lower time-averaged RMSE and BNorm values, and still maintains a good and stable performance at large bearing noise. Additionally, the PL-MMSE provides the comparable RMSE and BNorm performance to the best choice SAM-IVKF (i.e., κ=4) as shown in Figure 3. As mentioned above, the SAM-IVKF relies on the selection of the thresholds. When the SAM thresholds change (i.e., κ=3,2), there are visible degradations in the performance of the SAM-IVKF, as shown in Figure 4 and Figure 5, which is inferior to the proposed PL-MMSE. The selection of the SAM threshold depends on experience and there is no guarantee that the best threshold can be selected in practical applications. As a contrast, the PL-MMSE is derived under the unified MMSE estimation framework and does not depend on experience to choose any parameter, so it has stable performance. The PL-MMSE handle the correlation problem implicitly under the MMSE framework and does not require bias compensation as performed in BC-PLKF, so its estimation accuracy still can be guaranteed at large bearing noise level. Figure 6, Figure 7 and Figure 8 show the RMSEs and BNorms of the target position and velocity estimates versus time instant *k* for $\sigma_{\theta }=7^{\circ }$ with threshold κ=4,3 and 2, respectively. Similar results can be found in Figure 6, Figure 7 and Figure 8 as in Figure 3, Figure 4 and Figure 5.

For the purposes of computational complexity comparison, the methods are executed on the same platform and their averaged runtimes are presented in Table 2. For convenience, the averaged runtimes of the BC-PLKF, the SAM-IVKF and the PL-MMSE are normalized by that of the PLKF.

In Table 2, it can be seen that the PLKF has the lowest computational complexity, but it provides an unsatisfactory tracking performance in both small and large bearing noise levels. The BC-PLKF and SAM-IVKF have 18% and 71% longer runtimes compared to the PLKF due to the extra time to perform bias compensation steps. Similarly, the proposed PL-MMSE requires 46% longer runtimes than the PLKF to handle the correlation problems. Compared with the PLKF and the BC-PLKF, although sacrificing a bit of computational complexity, the PL-MMSE significantly outperforms the two methods in terms of the RMSE and BNorm performance. In addition, the SAM-IVKF requires larger computational complexity than the proposed PL-MMSE, and can only provide performance comparable to the PL-MMSE when selecting the best SAM threshold. Accordingly, the PL-MMSE is superior to the SAM-IVKF in both computational complexity and estimation accuracy.

**Figure 9:**
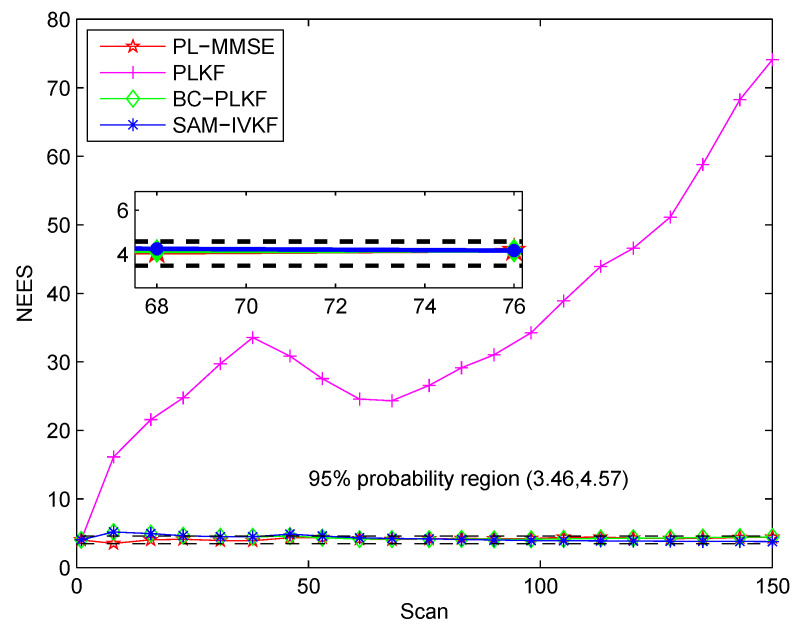
Consistency test of the PLKF, BC-PLKF, SAM-IVKF and the proposed PL-MMSE.

In the following, the consistency of the methods is examined based on the evaluation of the NEES. The bearing noise standard deviation is $\sigma_{\theta }=7^{\circ }$, and the SAM threshold of the SAM-IVKF is κ=4. Here, we use the two-sided 95% probability region, where the upper and lower bounds are 3.46 and 4.57, respectively. The NEES results of the four methods are presented in Figure 9.

The results in Figure 9 show the inconsistency of the PLKF since its NEES values are outside the region of 95%. The NEES values of the BC-PLKF, SAM-IVKF and PL-MMSE all fall within the 95% probability region, which indicates that the three methods are consistent. Among these methods, the proposed PL-MMSE can provide the most stable and accurate tracking performance.

## 6. Conclusions

In this paper, a new pseudolinear filtering method is proposed under the MMSE framework to solve the bearings-only target tracking problem. The proposed PL-MMSE does not require performing bias compensation to solve the correlation problem of PLKF. Instead, the PL-MMSE analyzes the correlation between the pseudomeasurement matrix and the pseudolinear noise, and their cross-covariance is incorporated into the filtering process under the MMSE framework to account for their effects on estimation. Accordingly, the correlation problem can be handled implicitly in the filtering process of the PL-MMSE. Simulations show that the PL-MMSE meets the consistency requirement and can provide stable and accurate tracking performance, which is superior to the PLKF and BC-PLKF at both small and large bearing noise levels. Additionally, the PL-MMSE is comparable to the best choice SAM-IVKF, and is better than the SAM-IVKF in terms of computational complexity. These results verify the effectiveness of the proposed PL-MMSE to solve the bearings-only target tracking problem.

## Figures and Tables

**Table 1:** The bearing noise standard deviation and initialization error in each level.

Level	1	2	3	4	5	6	7	8	9	10
$\sigma_{\theta }$ (degree)	1	2	3	4	5	6	7	8	9	10
ρ	1	2	3	4	5	6	7	8	9	10

**Table 2:** Averaged runtimes.

Algorithm	PLKF	BC-PLKF	SAM-IVKF	PL-MMSE
Runtime	1	1.18	1.71	1.46

## Data Availability

Not applicable.

## Abbreviations

The following abbreviations are used in this manuscript:

PLKF	Pseudolinear Kalman filter
MMSE	Minimum mean square error
PCRLB	Posterior Cramer–Rao Lower Bound
BOT	Bearings-only tracking
EKF	Extended Kalman filter
MPEKF	Modified polar coordinate extended Kalman filter
UKF	Unscented Kalman filter
PF	Particle filter
KF	Kalman filter
MPKF	Modified pseudolinear estimator
BC-PLKF	Bias-compensated pseudolinear Kalman filter
IV	Instrumental variable
IVKF	Instrumental variable Kalman filter
SAM	Selective-angle-measurement
SAM-IVKF	Selective-angle-measurement instrumental variable Kalman filter
2D	Two-dimensional
NCV	Nearly constant velocity
PL-MMSE	Pseudolinear-MMSE
RMSEs	Root mean square errors
BNorms	Bias Norms
NEES	Normalized estimation error squared
